# Supplementary material for: Maximizing meiotic crossover rates reveals the map of Crossover Potential
Source: Nat Commun. 2025 Jun 12;16:5306. doi: 10.1038/s41467-025-60663-y (PMC12162847; doi:10.1038/s41467-025-60663-y)
Supplement: Supplementary file 1 — Supplementary Information [file 41467_2025_60663_MOESM1_ESM.pdf]

Genotype sex (Col/Ler F1)	Number of cells	Total number of MLH1 foci	Total number of HEI10 foci	Total number of MLH1/HEI10 cofoci	% MLH1 colocalization with HEI10	% HEI10 colocalization with MLH1	Co-foci/cell
wild type female	12	86	83	82	95,3	98,8	6,8
wild type male	28	300	290	290	96,7	100,0	10,4
zyp1 female	12	150	147	147	98,0	100,0	12,3
zyp1 male	27	344	342	342	99,4	100,0	12,7
HEI10oe female	17	204	202	202	99,0	100,0	11,9
HEI10oe male	34	627	626	624	99,5	99,7	18,4
recq4 female	21	147	147	147	100,0	100,0	7,0
recq4 male	19	186	186	186	100,0	100,0	9,8
zyp1 HEI10oe female	26	817	789	774	94,7	98,1	29,8
zyp1 HEI10oe male	26	774	762	761	98,3	99,9	29,3
recq4 HEI10oe female	13	156	156	156	100,0	100,0	12,0
recq4 HEI10oe male	27	508	516	505	99,4	97,9	18,7
zyp1 recq4 female	15	197	188	187	94,9	99,5	12,5
zyp1 recq4 male	23	291	291	291	100,0	100,0	12,7
zyp1 recq4 HEI10oe female	22	426	414	412	96,7	99,5	18,7
zyp1 recq4 HEI10oe male	67	1264	1230	1195	94,5	97,2	17,8

Table S1. Analysis of MLH1-HEI10 foci co-localization at diplotene-diakinesis

Raw data can be found in Source_data_Figure2_HEI10 MLH1 counting - coller.xlsx

lab stock	Mutants	PCR WT primer	PCR mutant primer	size wt band	size mutant band
K#288	*zyp1-1 (col)*	288A+288B Digestion MboI		182bp+128bp	204bp+128bp
K#293	*zyp1-6 (ler)*	288A+288B	292A+292B	503bp	1273bp
K#231	*recq4a-4 (col)*	158A+158B	158B+K5GABI_o8409	900bp	600bp
	recq4b-2 (col)	230A+230B	230C+K3_LB-Salk2	1000bp	800bp
K#249	*recq4a-W387X (ler)*	249A+249B Digestion ScrF1		150bp	180bp
K#133	*mus81-2 (col)*	133A+133B	133B+K3_LB-Salk2	1000bp	750bp
K#166	*figl1-19 (col)*	166C+166D	166C+K3_LB-Salk2	675bp	800bp
K#198	*figl1-12 (Ler)*	198A+198B Digestion Pfe1		253bp+71bp+45bp	298bp+71bp
K#244	HEI10oe (C2) (Col)	244E+F	244A+B	496pb	1000pb

	identification primer	PCR product size
Col/ler polylmorphism	NGA151F+R	Col=160bp Ler =120bp

Primer name	Sequence(5'-3')
288A	ATAGATCGATTTCGTCATCT
288B	GAGGTGAAATATGAATCTGCT
292A	TCCAGTTATTACCCTGTTTCTGT
292B	AGTGAGGATGATCGGCCATT
158A	ATCAGAGCCACTCATTGTTG
158B	GTCCTGATCGTGTTGGACAG
K5_GABI_o8409	ATATTGACCATCATACTCATTGC
249A	GACCAAGGCAGATATGCCTGTGATACCTG
249B	GTCCAGGGAAATTCACGACTGCTC
230A	TCAGAAAGTTGCTCTGCGTC
230B	ACCAAGACCCTGCATATTGC
230C	ACTAGAGATACTTCAGGAGCTGAGC
K3_LB-Salk2	GCTTTCTTCCCTTCCTTTCTC
133A	CATGCTGACAGTTGAAGGTC
133B	CCTCAAACGTTTCTCCAAAT
244A	TATTTCTGTGTCTTGAGCTTAGTGC
244B	CAGTCACGACGTTGTAAAACGACG
244E	TAATGTAATAAAATAAGAACC
244F	ACTCTTTCATCTAATTTCTTC
NGA151F	GTTTTGGGAAGTTTTGCTGG
NGA151R	CAGTCTAAAAGCGAGAGTATGATG
198A	GCATCTTTCACCAGGTTCTTC
198B	TGGTAAAGAATGATAAACAAA
166C	AAGGGCAGAGACAGGCATAG
166D	CCGTGCCTCTGATGAATGTG

Table S2. Genotyping primers

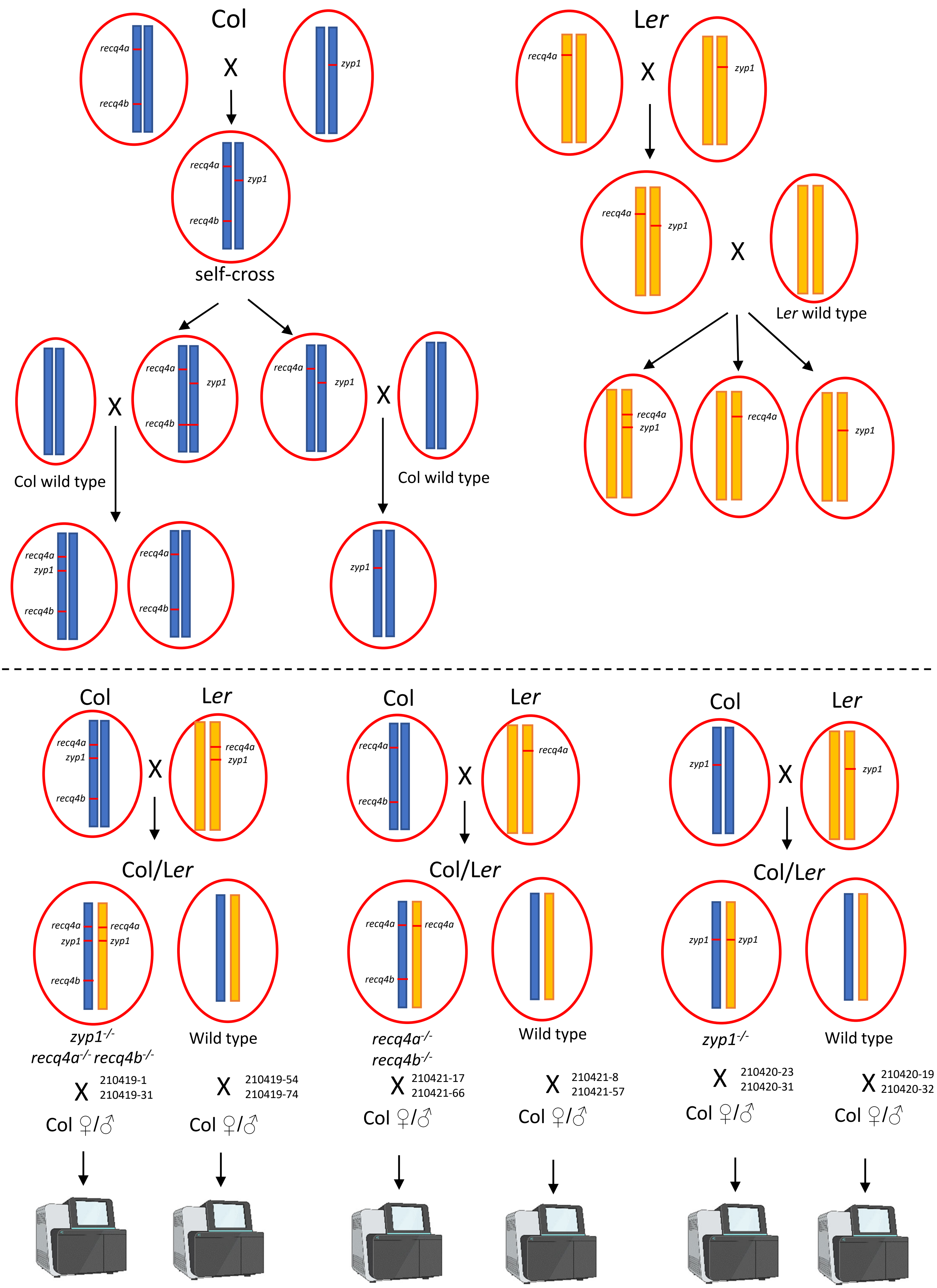
Col
X
recq4a
recq4b
zyp1
self-cross
Col wild type
Ler
Ler wild type
Col/Ler
zyp1-/- recq4a-/- recq4b-/-
Wild type
recq4a-/- recq4b-/-
zyp1-/-
210419-1
210419-31
210419-54
210419-74
210421-17
210421-66
210421-8
210421-57
210420-23
210420-31
210420-19
210420-32
Col ♀/♂

Fig S1.

Fig S1. Generation of *zyp1*$^{-/-}$ *recq4a*$^{-/-}$ *recq4b*$^{-/-}$(Col/L*er*), *recq4a*$^{-/-}$ *recq4b*$^{-/-}$ (Col/L*er*), *zyp1*$^{-/-}$ (Col/L*er*) and their respective wild type (Col/L*er*) controls.

zyp1$^{+/-}$(Col) plants were crossed with *recq4a*$^{+/-}$ *recq4b*$^{+/-}$*(*Col) plants to get *zyp1*$^{+/-}$ *recq4a*$^{+/-}$ *recq4b*$^{+/-}$ (Col), and then self-crossed to get *zyp1*$^{+/-}$ *recq4a*$^{+/-}$ *recq4b*$^{-/-}$*(*Col) and *recq4a*$^{+/-}$ *zyp1*$^{+/-}$(Col) plants. Note that ZYP1, RECQ4A and RECQ4B are all located on chromosome 1, to make sure the mutation of the three genes are all on the same chromosome , *zyp1*$^{+/-}$ *recq4a*$^{+/-}$ *recq4b*$^{-/-}$(Col. *zyp1*, *recq4a*, *recq4b* mutation in trans) were back crossed with Col wild type to get *zyp1*$^{+/-}$ *recq4a*$^{+/-}$ *recq4b*$^{+/-}$*(*Col. zyp1, recq4a and recq4b in cis) and *recq4a*$^{+/-}$ *recq4b*$^{+/-}$(Col. *recq4a*, *recq4b* in cis); *recq4a*$^{+/-}$ *zyp1*$^{+/-}$ (Col, *recq4a* and *zyp1* in trans) were crossed with Col wild type to get zyp1$^{+/-}$(Col). *zyp1*$^{+/-}$(L*er*) were crossed with *recq4a*$^{+/-}$(L*er*) to get *zyp1*$^{+/-}$ *recq4a*$^{+/-}$(L*er, zyp1* and recq4 in trans), then *zyp1*$^{+/-}$ *recq4a*$^{+/-}$ (L*er*, *zyp1* and *recq4* in trans) were backcrossed with wild type (L*er*) to get *zyp1*$^{+/-}$ *recq4a*$^{+/-}$(L*er*, *zyp1* and *recq4* in cis), *recq4a*$^{+/-}$(L*er*) and *zyp1*$^{+/-}$(L*er*). Then *zyp1*$^{+/-}$ *recq4a*$^{+/-}$ *recq4b*$^{+/-}$ (Col, *zyp1*, *recq4a* and *recq4b* in cis) were crossed with *zyp1*$^{+/-}$ *recq4a*$^{+/-}$ (L*er*, *zyp1* and *recq4a* are in cis) to get zyp1$^{-/-}$ recq4a$^{-/-}$ recq4b$^{-/-}$(Col/L*er*) and sister wild type(Col/L*er*) control; *recq4a*$^{+/-}$ *recq4b*$^{+/-}$ (Col) were crossed with *recq4a*$^{+/-}$ (L*er*) to get *recq4a*$^{-/-}$ *recq4b*$^{-/-}$ (Col/L*er*) and sister wild type(Col/L*er*) control; *zyp1*$^{+/-}$(Col) were crossed with *zyp1*$^{+/-}$(L*er*) plants to get *zyp1*$^{-/-}$(Col/L*er*) and sister wild type(Col/L*er*) control. Created in BioRender. Lian, Q. (2025) https://BioRender.com/m7vjhfg

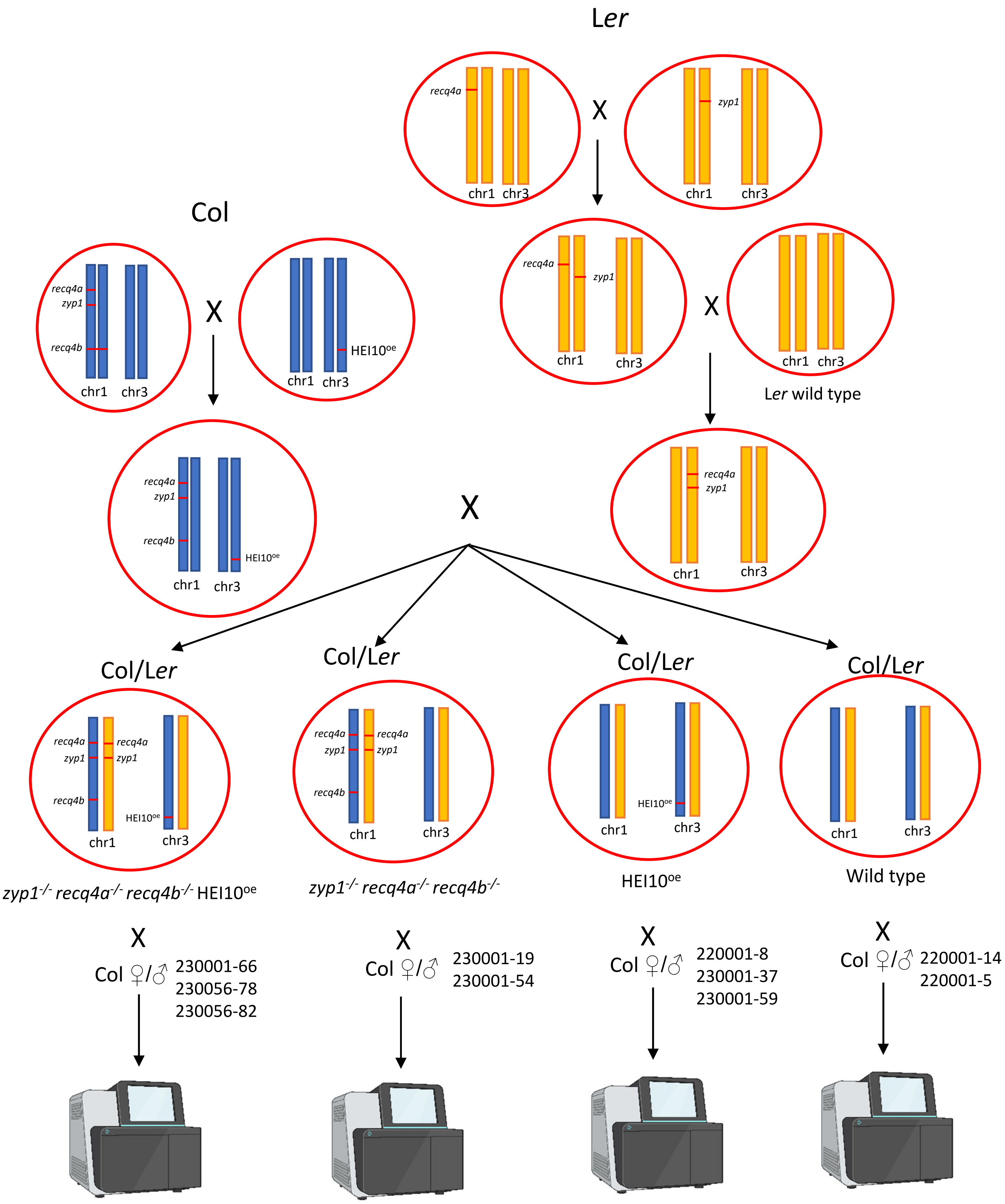

Fig S2. Generation of *zyp1*$^{-/-}$ *recq4a*$^{-/-}$ *recq4b*$^{-/-}$ HEI10^{oe} (Col/*Ler*), *zyp1*$^{-/-}$ *recq4a*$^{-/-}$ *recq4b*$^{-/-}$ (Col/*Ler*), HEI10^{oe} (Col/*Ler*) and wild type (Col/*Ler*)

zyp1$^{+/-}$ *recq4a*$^{+/-}$ *recq4b*$^{-/-}$ (Col) were crossed with HEI10oehz (Col) to get *zyp1*$^{+/-}$ *recq4a*$^{+/-}$ *recq4b*$^{+/-}$ HEI10oehz (Col. *zyp1*, *recq4a* and *recq4b* in cis), *then zyp1*$^{+/-}$ *recq4a*$^{+/-}$ *recq4b*$^{+/-}$ HEI10oehz (Col. *zyp1*, *recq4a* and *recq4b* in cis) were crossed with *zyp1*$^{+/-}$ *recq4a*$^{+/-}$(*Ler*, zyp1 and *recq4a* in cis) to get *zyp1*$^{-/-}$ *recq4a*$^{-/-}$ *recq4b*$^{-/-}$ HEI10oehz (Col/*Ler*), *zyp1*$^{-/-}$ *recq4a-/-* *recq4b*$^{-/-}$ (Col/*Ler*), HEI10oehz (Col/*Ler*) and wild type. Created in BioRender. Lian, Q. (2025) https://BioRender.com/m7vjhfg.

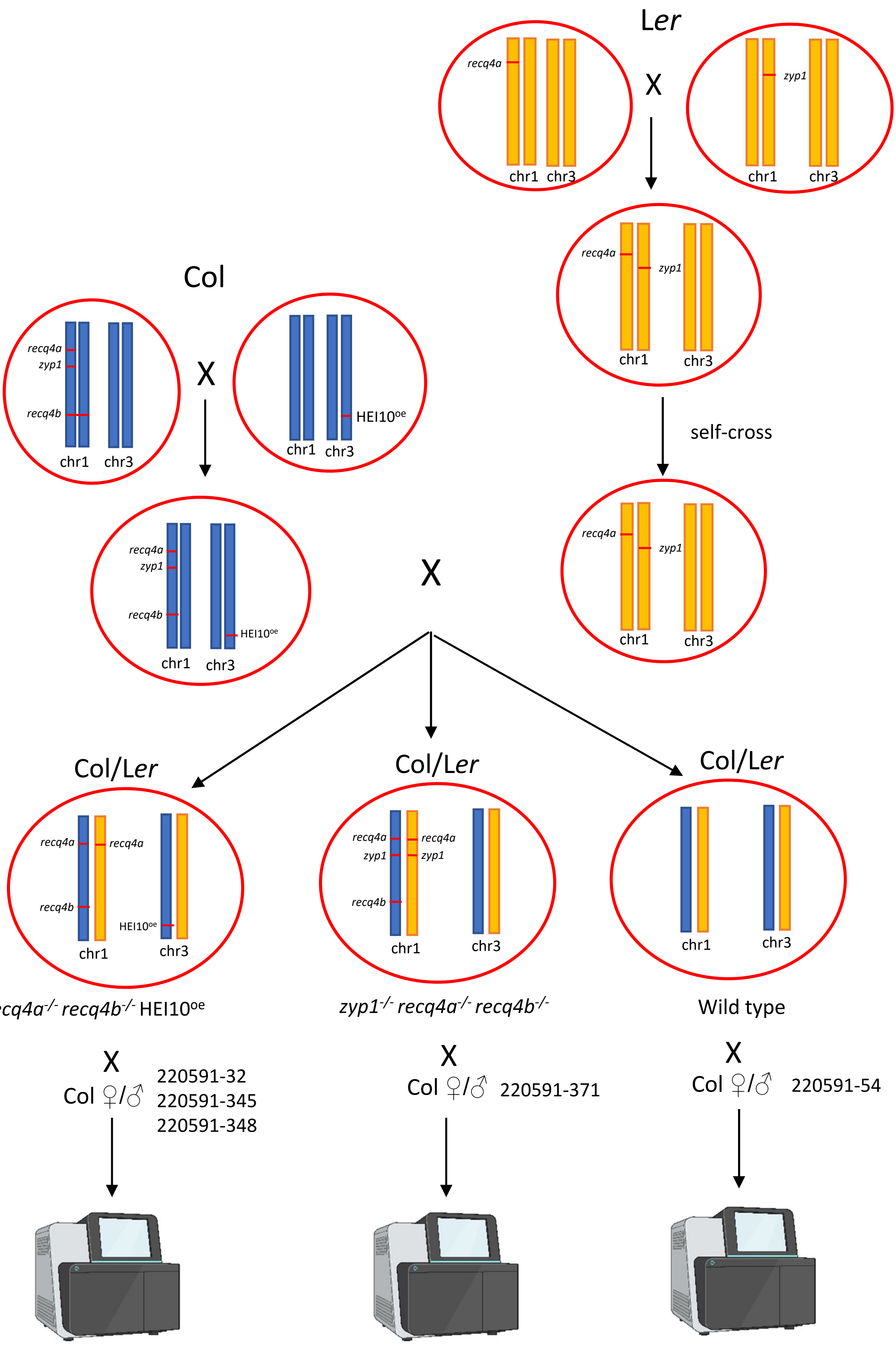

Fig S3. Generation of *recq4a$^{-/-}$ recq4b$^{-/-}$* HEI10^{oe} (Col/L*er*), *zyp1$^{-/-}$ recq4a$^{-/}$ recq4b$^{-/-}$* (Col/L*er*), wild type (Col/L*er*).

zyp1$^{+/-}$ recq4a$^{+/-}$ recq4b$^{-/-}$ (Col) were crossed with HEI10oehz (Col) to get *zyp1$^{+/-}$ recq4a$^{+/-}$ recq4b$^{+/-}$* HEI10oehz (Col. *zyp1*, *recq4a* and *recq4* in cis), *zyp1$^{+/-}$ recq4a$^{+/-}$ recq4b$^{+/-}$* HEI10oehz (Col, *zyp1*, *recq4a* and *recq4b* in cis) were crossed with *zyp1$^{+/-}$ recq4a$^{+/-}$* (L*er*, *zyp1* and *recq4a* in trans) to get *recq4a$^{-/-}$ recq4b$^{-/-}$* HEI10oehz (Col/L*er*), *zyp1$^{-/-}$ recq4a$^{-/-}$ recq4b$^{-/-}$* (Col/L*er*) and wild type (Col/L*er*). Created in BioRender. Lian, Q. (2025) https://BioRender.com/m7vjhfg

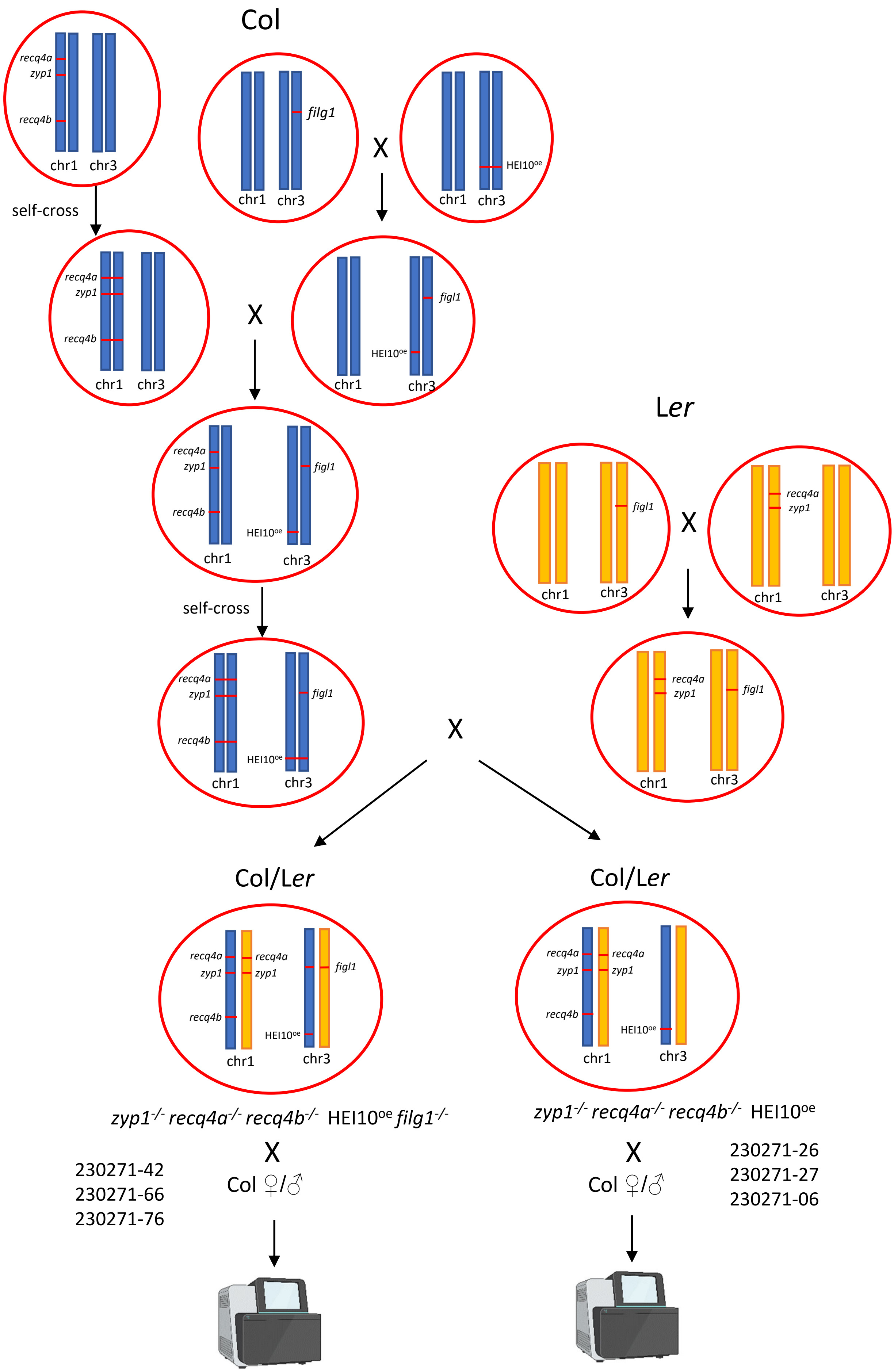
Col
recq4a
zyp1
recq4b
chr1
chr3
filg1
X
HEI10oe
self-cross
figl1
Ler
self-cross
Col/Ler
Col/Ler
zyp1-/- recq4a-/- recq4b-/- HEI10oe filg1-/-
zyp1-/- recq4a-/- recq4b-/- HEI10oe
X
Col ♀/♂
230271-42
230271-66
230271-76
230271-26
230271-27
230271-06

Fig S4.

Fig S4. Generation of *zyp1*$^{-/-}$ *recq4a*$^{-/-}$ *recq4b*$^{-/-}$ HEI10^{oe} *figl1*$^{-/-}$ (Col/L*er*), and *zyp1*$^{-/-}$ *recq4a*$^{-/-}$ *recq4b*$^{-/-}$ HEI10^{oe} (Col/L*er*).

zyp1$^{+/-}$ *recq4a*$^{+/-}$ *recq4b*$^{+/-}$(Col. *zyp1, recq4a* and *recq4b* in cis) were self-crossed to get *zyp1*$^{-/-}$ *recq4a*$^{-/-}$ *recq4b*$^{-/-}$ (Col), *figl1*$^{+/-}$(Col) were crossed with HEI10oe homo (Col) to get HEI10oehz *figl1*$^{+/-}$ (Col). Then *zyp1-/- recq4a-/- recq4b*$^{-/-}$ (Col) were crossed with HEI10oehz *figl1*$^{+/-}$(Col, HEI10oehz and *figl1* in trans) to get *zyp1*$^{+/-}$ *recq4a*$^{+/-}$ *recq4b*$^{+/-}$ HEI10oehz *figl1*$^{+/-}$(Col. *zyp1, recq4a* and *recq4b* in cis, HEI10oehz and *figl1* in trans we don't know), then this plant was self-crossed to get *zyp1-/- recq4a-/- recq4b-/-* HEI10oehomo *figl1*$^{+/-}$ (Col). *zyp1*$^{+/-}$ *recq4a*$^{+/-}$ (L*er*, *zyp1* and *recq4a* in cis) were crossed with *figl1*$^{+/-}$(L*er*) to get *zyp1*$^{+/-}$ *recq4a*$^{+/-}$ *figl1*$^{+/-}$ (L*er*, *zyp1* and *recq4a* in cis). Then *zyp1*$^{-/-}$ *recq4a*$^{-/-}$ *recq4b*$^{-/-}$ HEI10oehomo *figl1*$^{+/-}$ (Col) were crossed with *zyp1*$^{+/-}$ *recq4a*$^{+/-}$ *figl1*$^{+/-}$ (L*er*, zyp1 recq4a in cis) to get *zyp1*$^{-/-}$ *recq4a*$^{-/-}$ *recq4b*$^{-/-}$ HEI10oehz *figl1*$^{-/-}$ (Col/L*er*) and *zyp1*$^{-/-}$ *recq4a*$^{-/-}$ *recq4b*$^{-/-}$ HEI10oe hz (Col/L*er*) (Fig. S4). Created in BioRender. Lian, Q. (2025) https://BioRender.com/m7vjhfg.

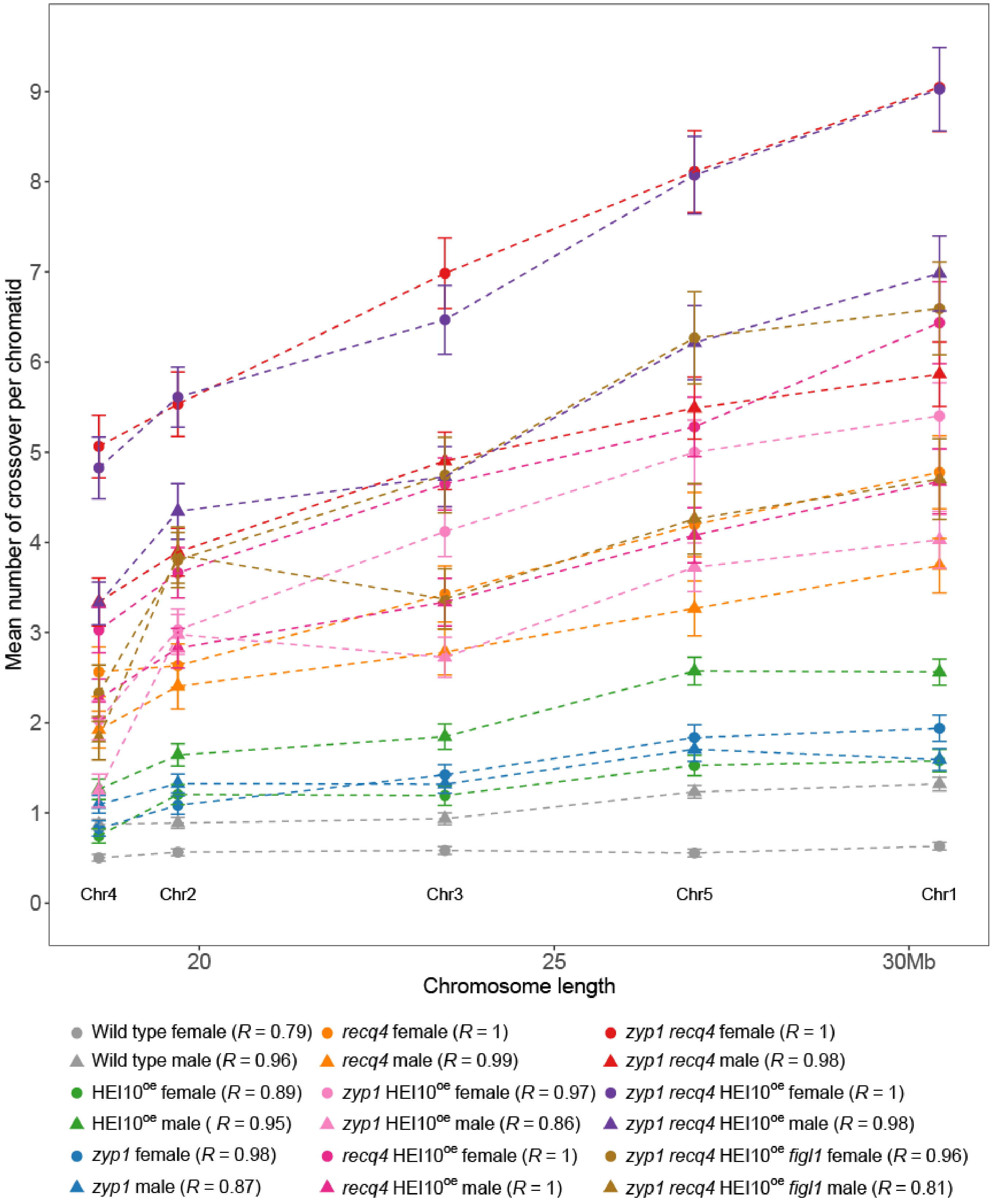

Fig S5. Correlation analysis between mean number of COs per transmitted chromatid and chromosome size.

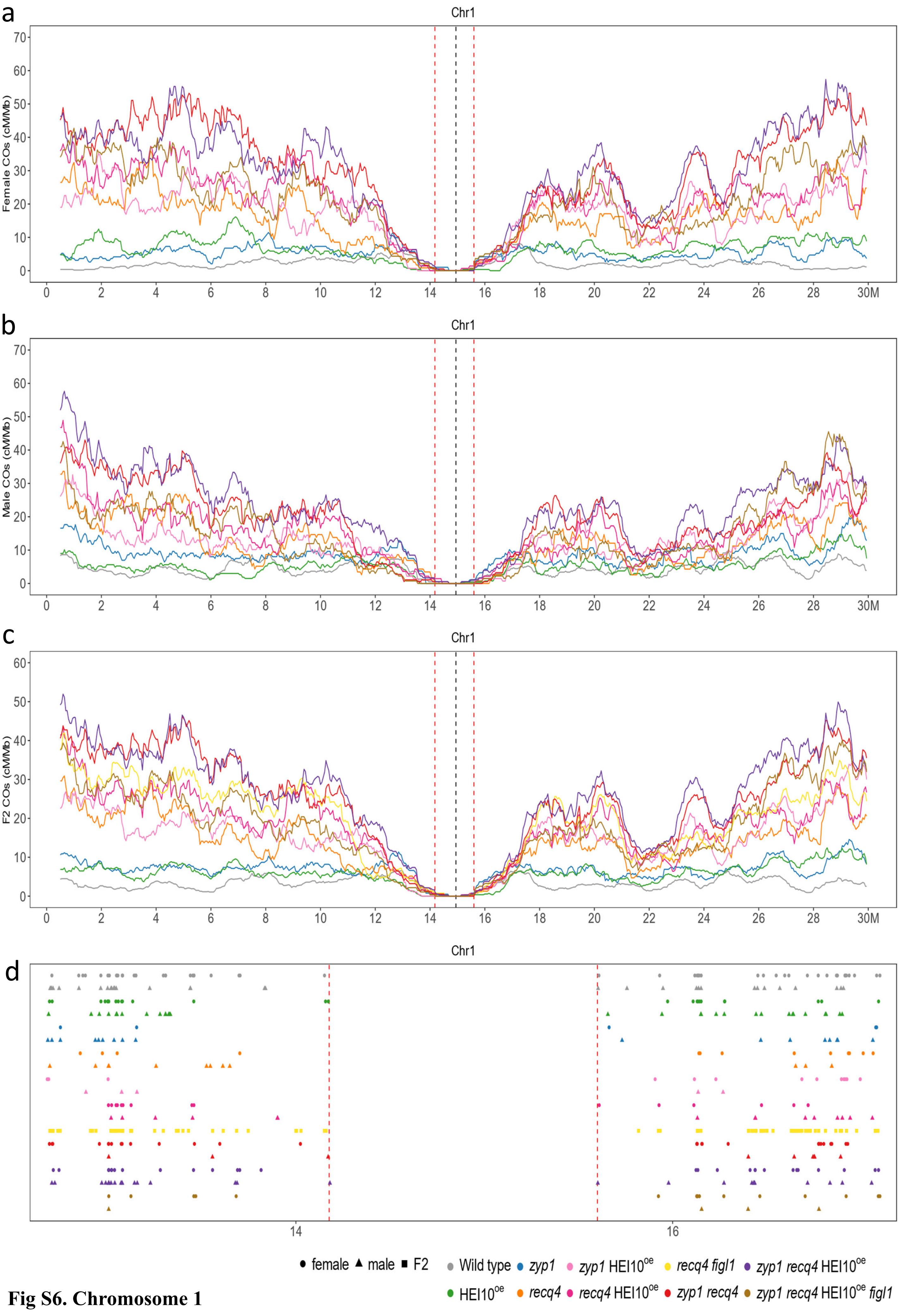

Fig S6. Chromosome 1

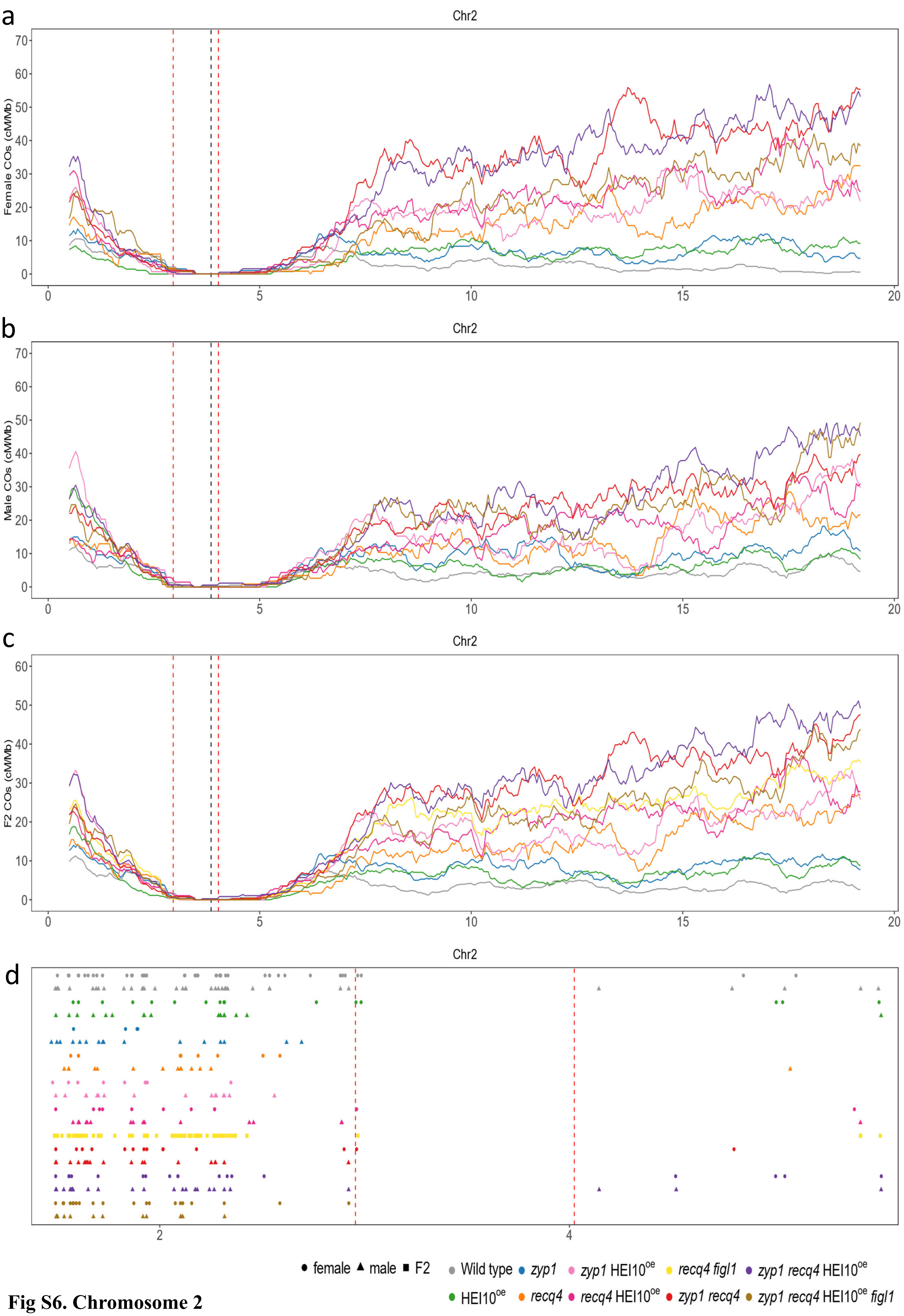

Fig S6. Chromosome 2

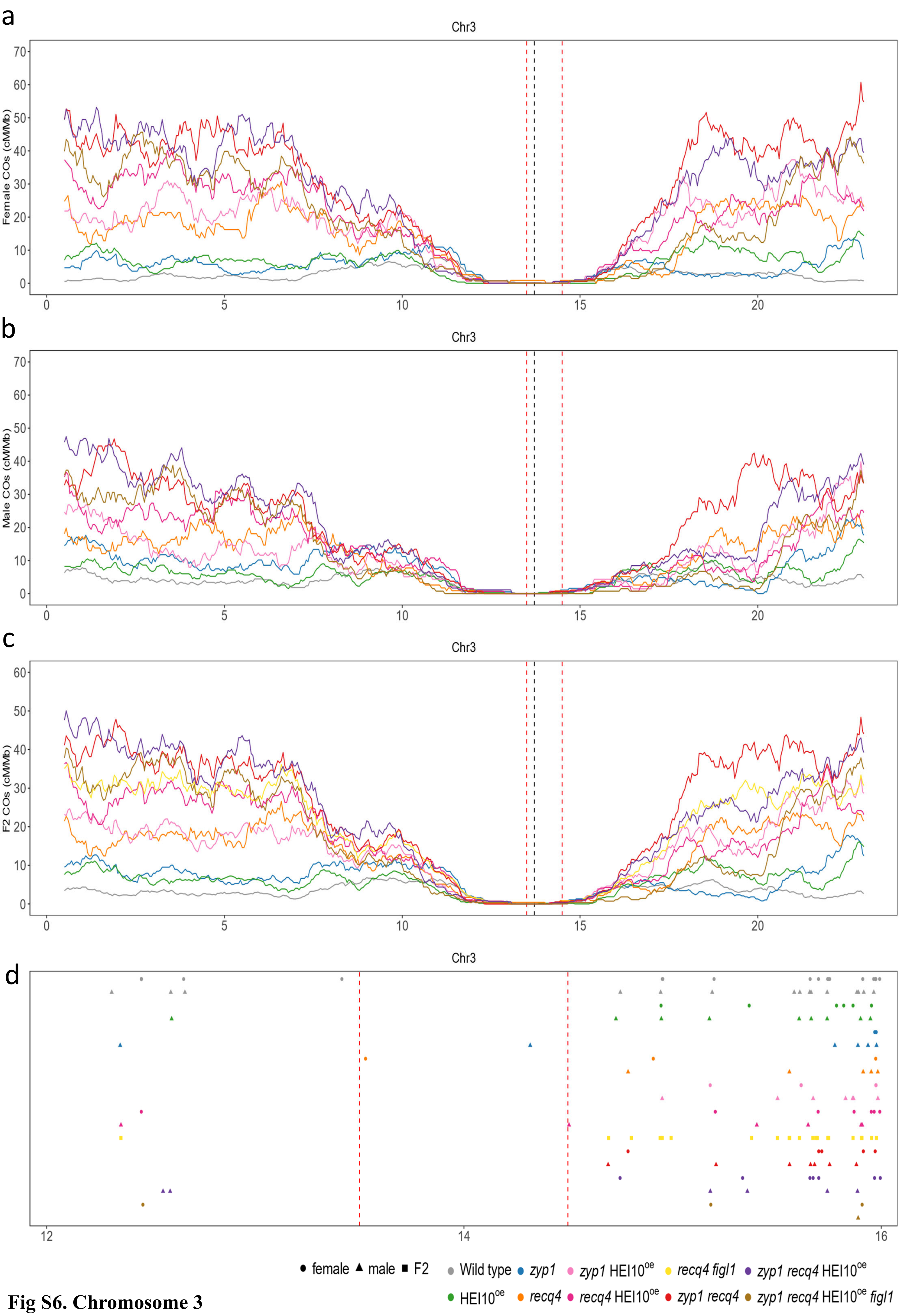

Fig S6. Chromosome 3

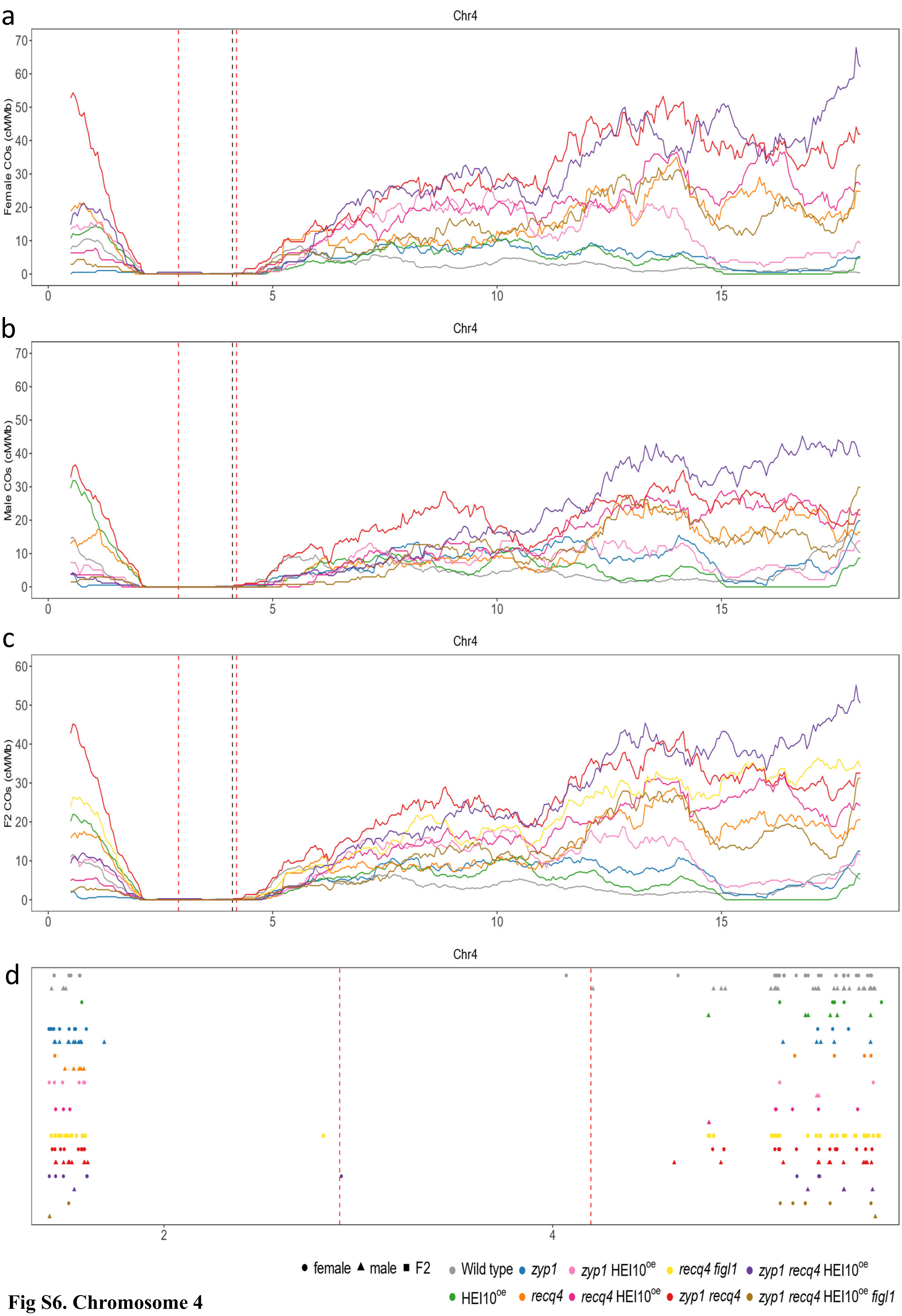

Fig S6. Chromosome 4

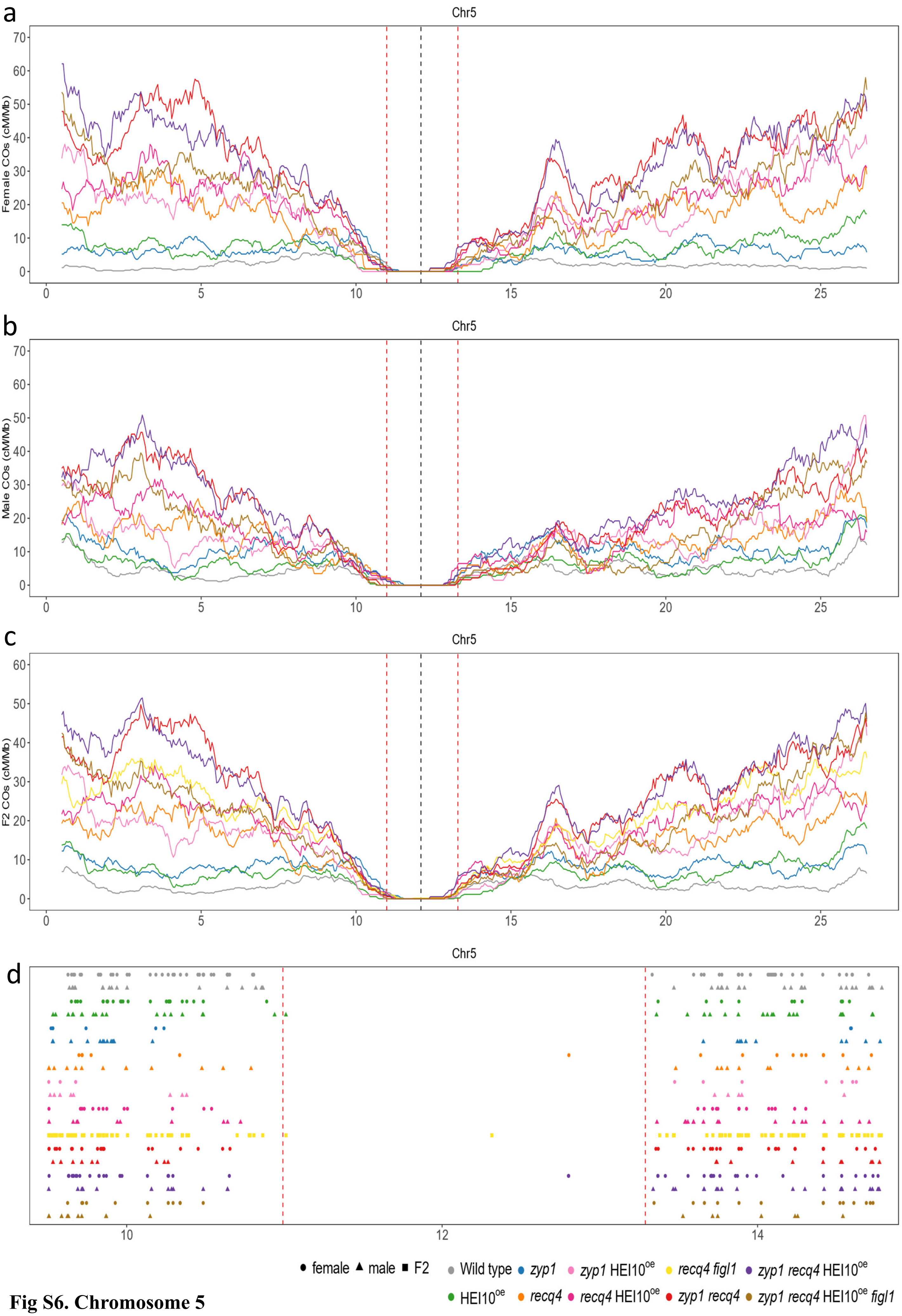

Fig S6. Chromosome 5

Figure S6. Chromosomal distribution of COs in female, male, and F2 contexts.

(zoom of figure 4)

a-b. The distribution (sliding window-based, window size 1 Mb, step size 50 kb) of COs along chromosomes in female (**a**) and male (**b**) of wild type, HEI10^{oe}, *zyp1*, *recq4*, *zyp1* HEI10^{oe}, *recq4* HEI10^{oe}, *zyp1 recq4*, *zyp1 recq4* HEI10^{oe} and *zyp1 recq4* HEI10^{oe} *figl1*. **c.** The distribution (sliding window-based, window size 1 Mb, step size 50 kb) of COs along chromosomes in F2 or pseudo F2 of wild type, HEI10^{oe}, *zyp1*, *recq4*, *zyp1* HEI10^{oe}, *recq4* HEI10^{oe}, *recq4 figl1*, *zyp1 recq4*, *zyp1 recq4* HEI10^{oe} and *zyp1 recq4* HEI10^{oe} *figl1*. **d.** The zoom of the CO position in the centromere proximal regions (Non-Recombining Zones, NRZs). Each point is a CO, circles, triangles and squares are females, males and F2s, respectively. The vertical dashed lines in red indicates the position of marker COs of NRZs, lines in black shows the middle position of centromeres

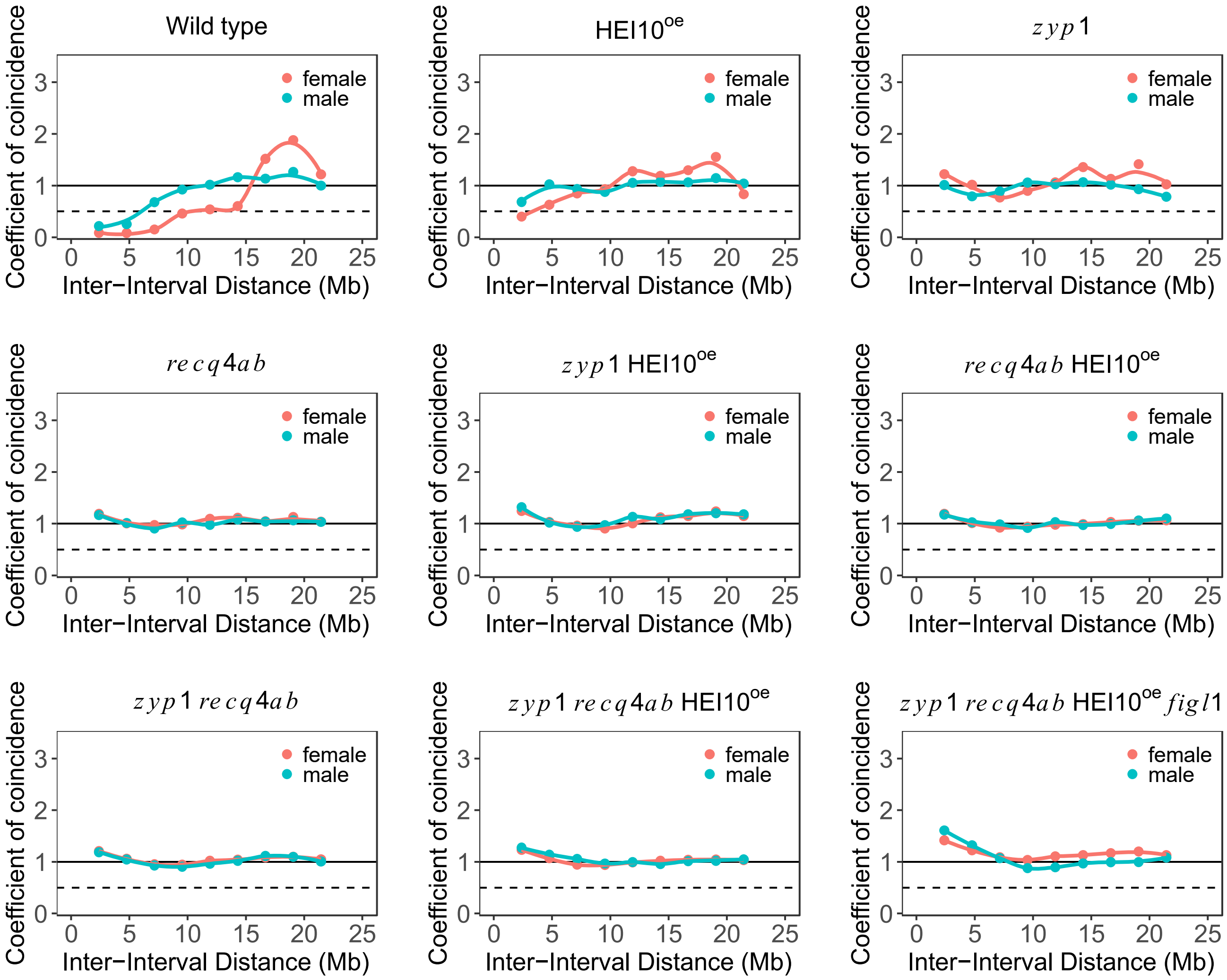

Fig S7. Analysis of CO interference.

CoC curves in the female and male meiosis of wild type, HEI10^{oe}, *zyp1*, *recq4*, *zyp1* HEI10^{oe}, *recq4* HEI10^{oe}, *zyp1 recq4*, *zyp1 recq4* HEI10^{oe} and *zyp1 recq4* HEI10^{oe} *figl1*, respectively. Chromosomes were divided into 10 intervals, for calculating the mean coefficient of coincidence of each pair of intervals. A CoC close to 1 means the absence of CO interference. A CoC close to 0 reveals an absence of double COs and is thus the presence of CO interference. The crossover interference is undetectable in both female and male meiosis of hyper recombination mutants with ZYP1 and/or RECQ4 mutations, consistent with previous studies (Séguéla-Arnaud et al. 2015; Serra et al. 2018; Fernandes et al. 2018; France et al. 2021; Capilla-Pérez et al. 2021; Durand et al. 2022).

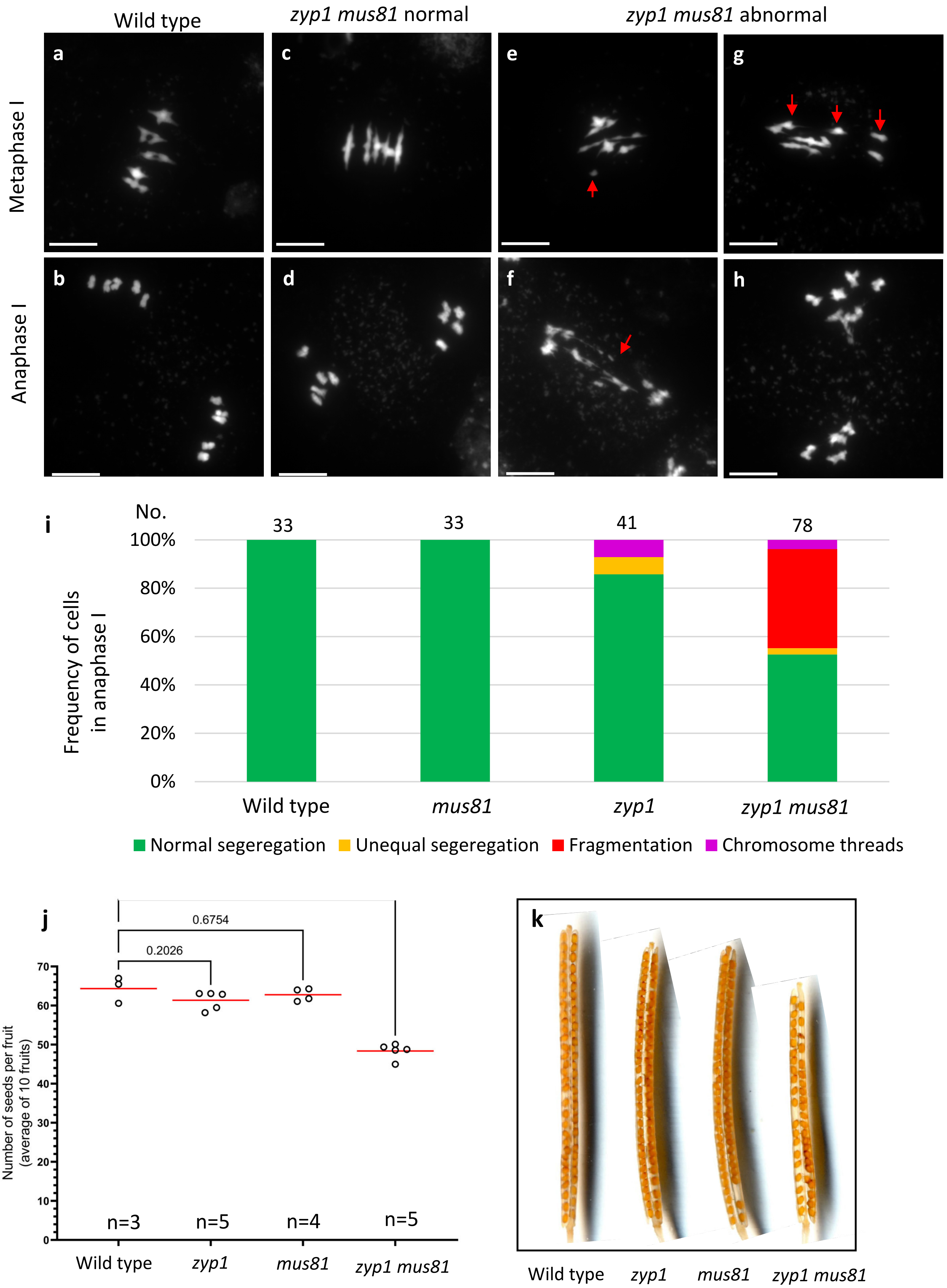
Wild type
zyp1 mus81 normal
zyp1 mus81 abnormal
Metaphase I
Anaphase I
a
b
c
d
e
f
g
h
i
No.
33
33
41
78
100%
80%
60%
40%
20%
0%
Frequency of cells in anaphase I
Wild type
mus81
zyp1
zyp1 mus81
Normal segeregation
Unequal segeregation
Fragmentation
Chromosome threads
j
0.6754
0.2026
70
60
50
40
30
20
10
0
Number of seeds per fruit (average of 10 fruits)
n=3
n=5
n=4
n=5
Wild type
zyp1
mus81
zyp1 mus81
k
Wild type
zyp1
mus81
zyp1 mus81

Fig S8.

Fig S8. Analysis of meiosis and fertility in wild type, *zyp1*, *mus81* and *zyp1 mus81*.

a-g. DAPI-stained meiotic chromosome spreads from male meiocytes in wild type (**a, b**), *zyp1*(**c, d**), *mus81* (**e,f**) and *zyp1 mus81* (**g, h**). **a, c, e, g** Metaphase I, **b, d, f, h** Anaphase I. Red arrows pointed out abnormal chromosome connections, fragments and chromosome threads. **i.** Quantification of different chromosome behaviors at metaphase I in wild type, *zyp1*, *mus81* and *zyp1 mus81* in Col background. Cells were categorized according to normal (5 bivalents) and abnormal chromosome behavior (unequal segregation, fragmentation and chromosome threads). The number of analyzed cells are indicated above the bar. **j.** Quantification of fertility. Each dot represents the fertility of an individual plant, measured as the number of seeds per fruits averaged on ten fruits. The red bar shows the mean. All plants were grown in parallel, and the wild-type controls are siblings of the mutants. The number n of analyzed plants is indicated and p values are one-way ANOVA followed by Fisher's LSD test. **k.** Representative cleared fruits of wild type, *zyp1*, *mus81* and *zyp1 mus81* in Col background.

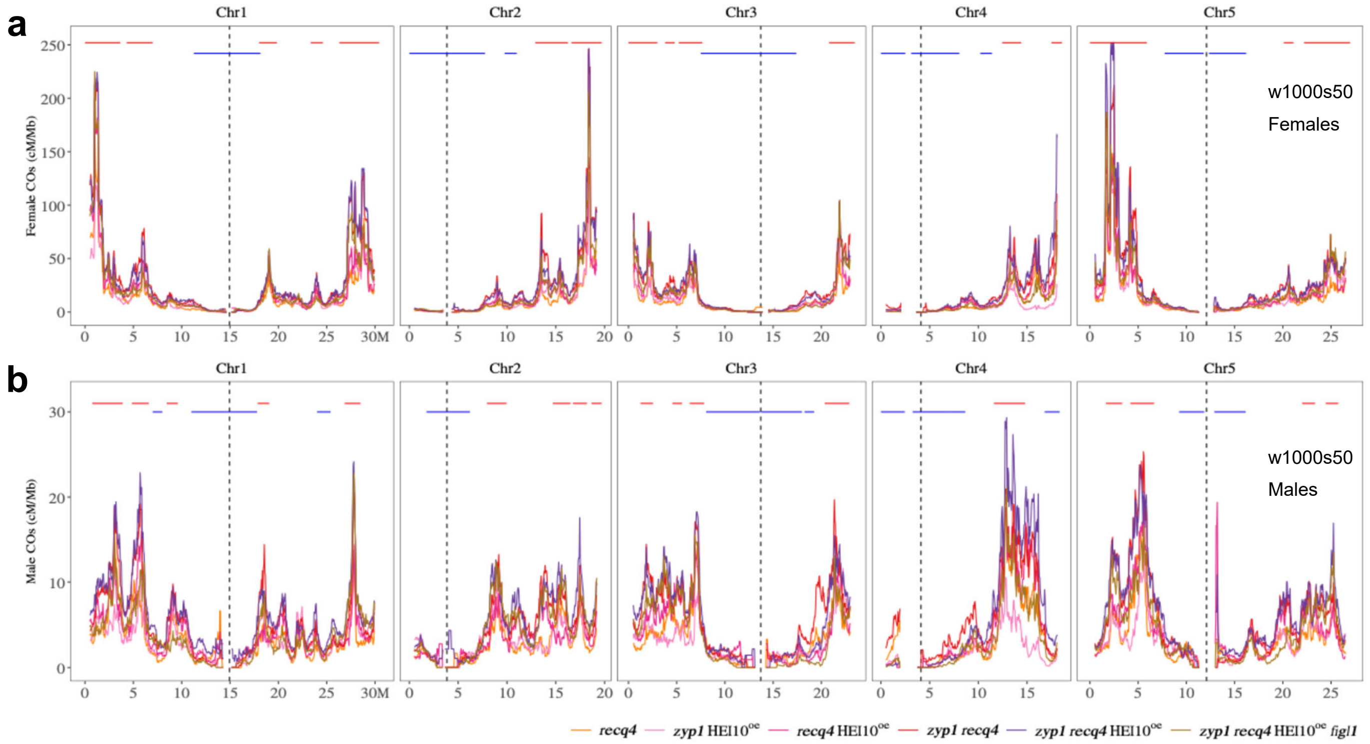

Fig S9. The fold-change analysis of CO frequencies along chromosomes in females and males.

The chromosomal distribution of fold-changes of CO frequencies (sliding window-based, window size 1 Mb, step size 50 kb) against wild-type in females (**a.**) and males (**b.**), respectively. The hot and cold zones of CO burst from hyper recombination mutants were colored by red and blue horizontal lines, separately.

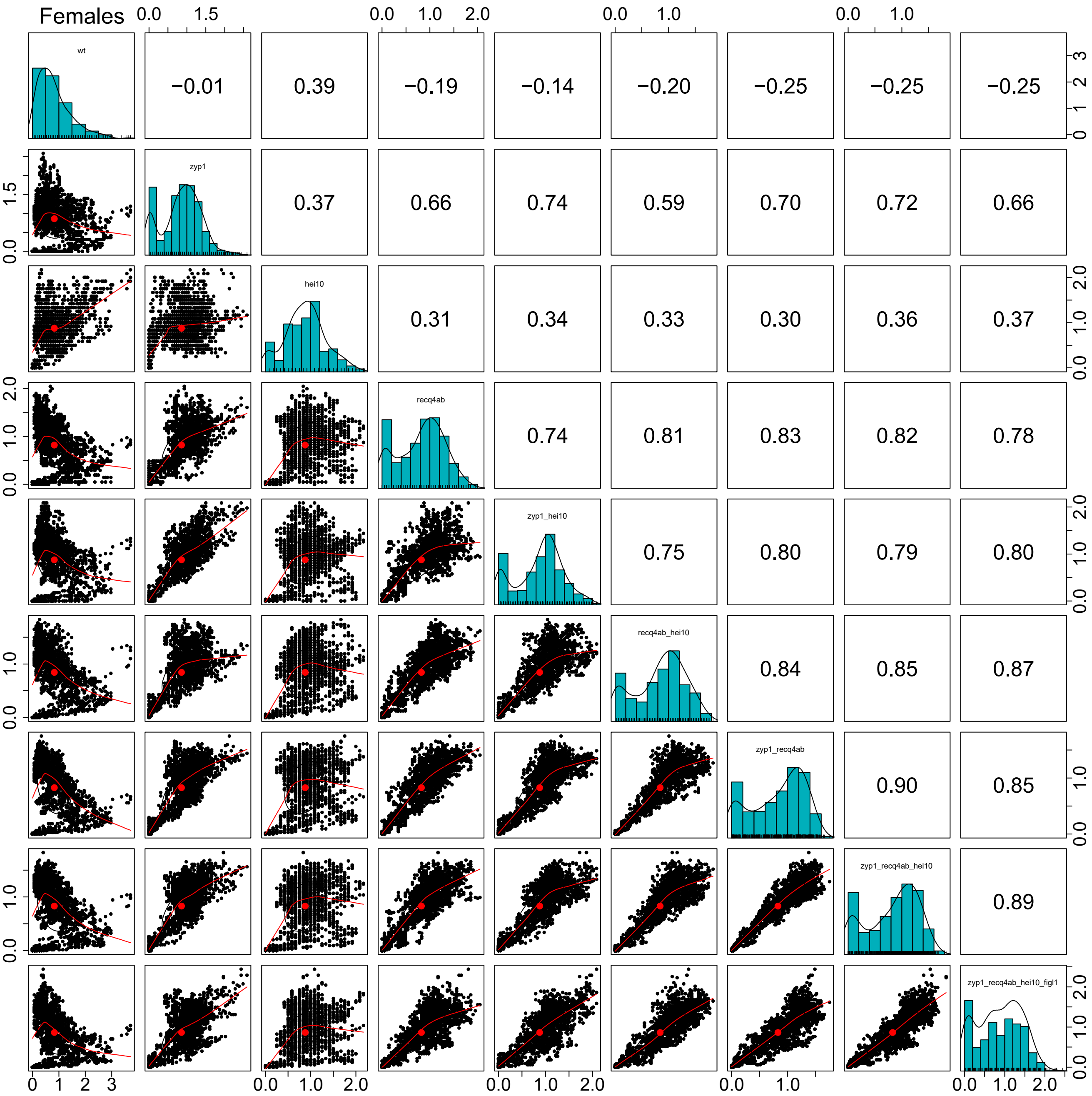

Fig S10. Correlation analysis of the relative distribution of COs in females.

Spearman's correlation between each pair of genotypes is shown in the upper triangle panel. The chromosome 4 is ignored in the analysis.

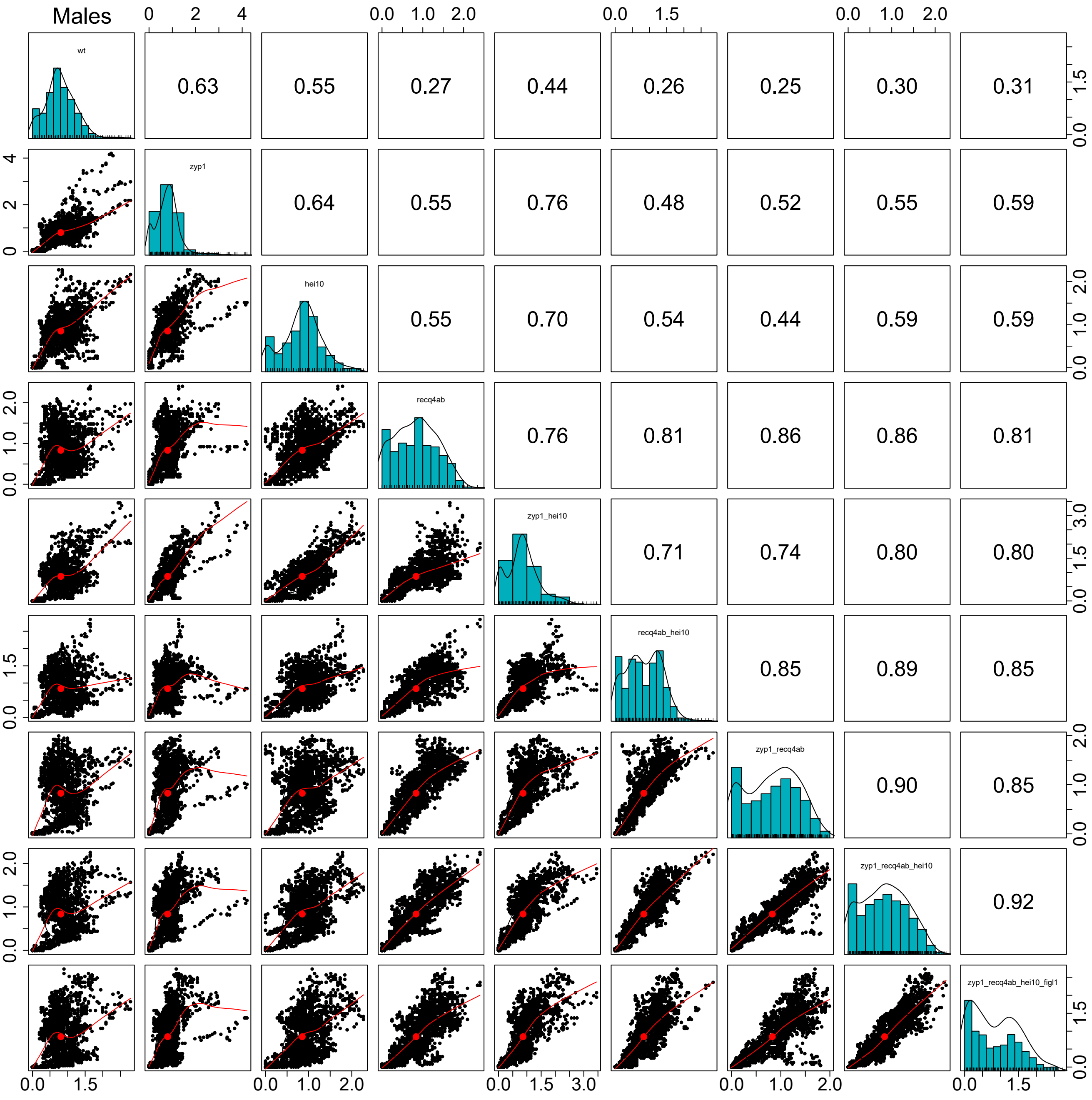

Fig S11. Correlation analysis of the relative distribution of COs in males.

Spearman's correlation between each pair of genotypes is shown in the upper triangle panel. The chromosome 4 is ignored in the analysis.

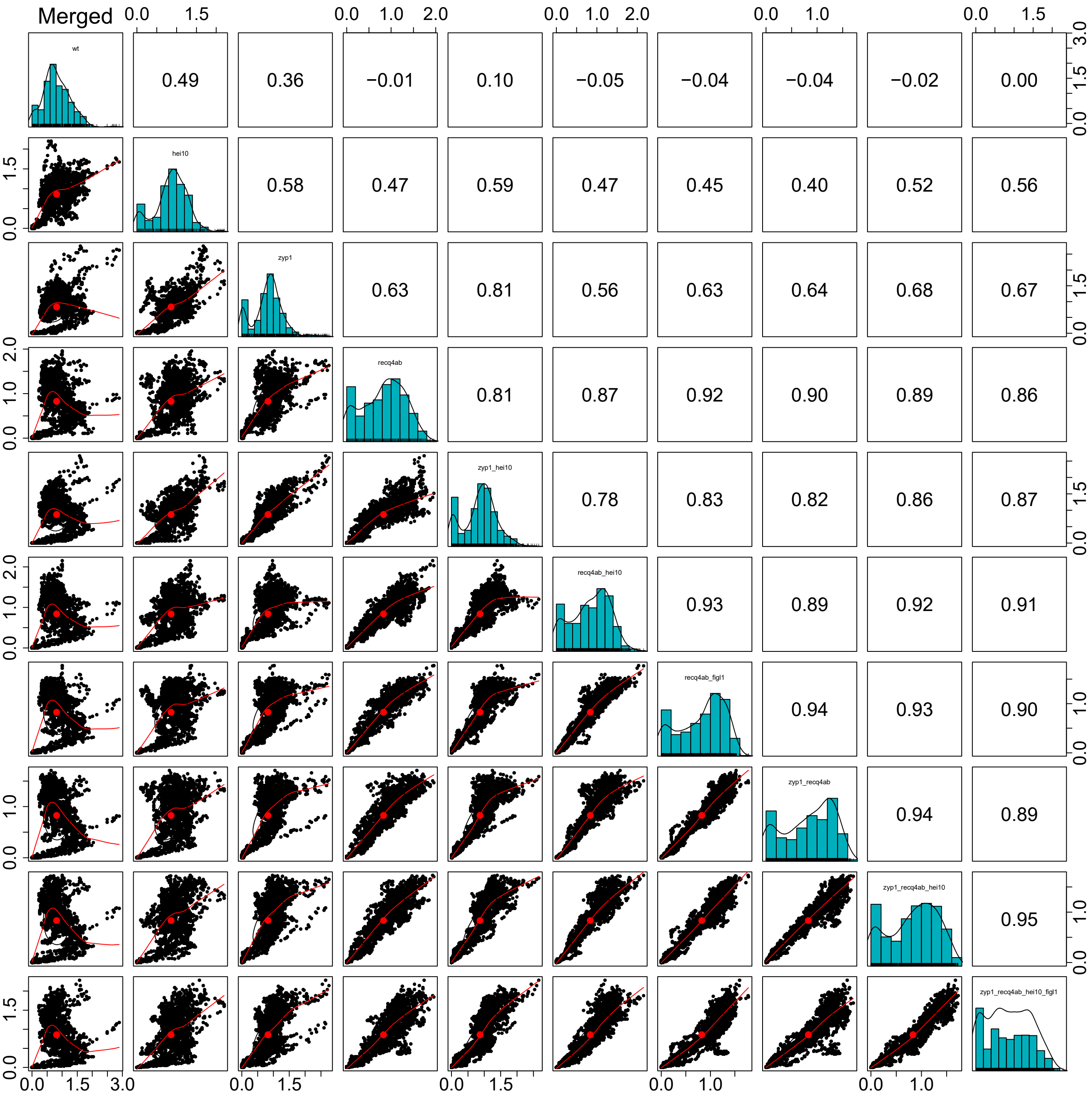

Fig S12. Correlation analysis of the relative distribution of COs in F2s or pseudo F2.

Spearman's correlation between each pair of genotypes is shown in the upper triangle panel. The chromosome 4 is ignored in the analysis.

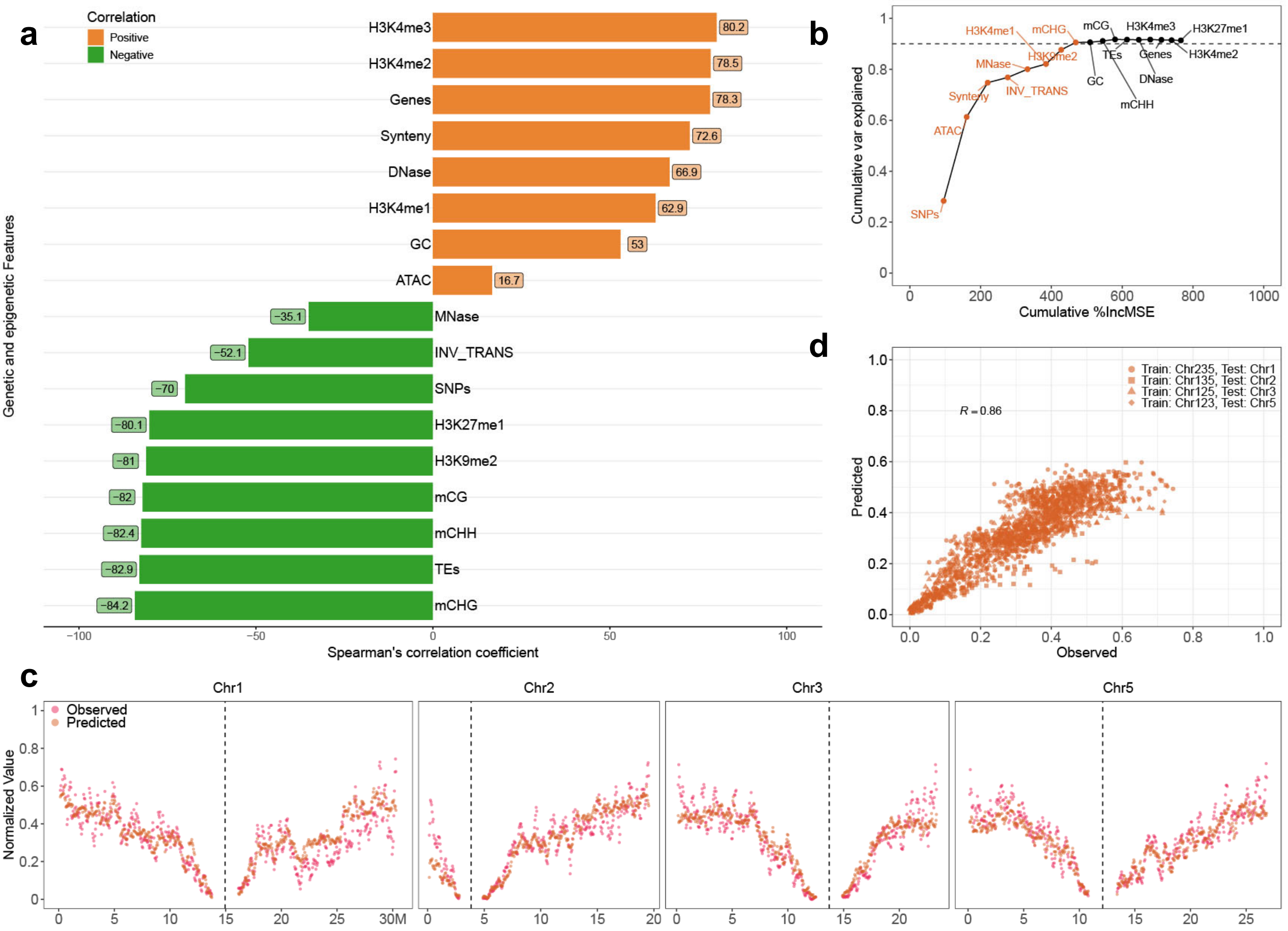

Fig S13. Association and prediction of precursor distribution with genetic and epigenetic features on chromosome arms.

a. Spearman's correlation test shows the comparison with features along chromosome arms, with differences in colour and length according to the correlation scale. SNPs (SNPs density between Col and L*er*), INV_TRANS (inversions and translocations between Col and L*er*), Synteny (collinearity between Col and L*er*), Genes, TEs and GC (expressed gene in meiocytes, TE and GC density), ATAC and DNase (chromatin accessibility, ATAC-seq and DNase-seq, log2(Tn5/gDNA) and log2(DNase/gDNA)), H3K4me1/2/3, H3K9me2, H3K27me1 (euchromatin, heterochromatin, and Polycomb histone marks, ChIP-seq, log2(ChIP/input)), mCG, mCHG and mCHH (DNA methylation in CG, CHG, and CHH contexts, proportion methylated cytosine), MNase (nucleosome occupancy, MNase-seq, log2(MNase/gDNA)). **b.** The cumulated proportion of variation that can be explained with the features at the chromosome arm scale. The top eight most important features are coloured, for which the cumulative proportion of variation that can be explained reaches the plateau. **c.** The chromosomal distribution of observed and predicted precursor maps. The precursor profiles of individual chromosomes were predicted using profiles of the top eight most important features from the other three chromosomes. **d.** The Spearman's correlation test between the predicted and observed precursor distributions. The training-testing dataset is differentiated in shapes.
