# Supplementary material for: Maximizing meiotic crossover rates reveals the map of Crossover Potential
Source: Nat Commun. 2025 Jun 12;16:5306. doi: 10.1038/s41467-025-60663-y (PMC12162847; doi:10.1038/s41467-025-60663-y)
Supplement: Supplementary file 3 — Description of Additional Supplementary Files [file 41467_2025_60663_MOESM3_ESM.pdf]

Description of Additional Supplementary Files

File Name: Supplementary Data 1
Description: List of crossovers and their position

File Name: Supplementary Data 2
Description: Complete Z-stack of the figure 2 images
