# Supplementary material for: Maximizing meiotic crossover rates reveals the map of Crossover Potential
Source: Nat Commun. 2025 Jun 12;16:5306. doi: 10.1038/s41467-025-60663-y (PMC12162847; doi:10.1038/s41467-025-60663-y)
Supplement: Supplementary file 6 — Reporting Summary [file 41467_2025_60663_MOESM6_ESM.pdf]

nature portfolio

Corresponding author(s): Raphael Mercier

Last updated by author(s): 2025/03/14

Reporting Summary

Nature Portfolio wishes to improve the reproducibility of the work that we publish. This form provides structure for consistency and transparency in reporting. For further information on Nature Portfolio policies, see our Editorial Policies and the Editorial Policy Checklist.

Statistics

For all statistical analyses, confirm that the following items are present in the figure legend, table legend, main text, or Methods section.

n/a	Confirmed	
☐	☒	The exact sample size (*n*) for each experimental group/condition, given as a discrete number and unit of measurement
☐	☒	A statement on whether measurements were taken from distinct samples or whether the same sample was measured repeatedly
☐	☒	The statistical test(s) used AND whether they are one- or two-sided<br>*Only common tests should be described solely by name; describe more complex techniques in the Methods section.*
☐	☒	A description of all covariates tested
☐	☒	A description of any assumptions or corrections, such as tests of normality and adjustment for multiple comparisons
☐	☒	A full description of the statistical parameters including central tendency (e.g. means) or other basic estimates (e.g. regression coefficient) AND variation (e.g. standard deviation) or associated estimates of uncertainty (e.g. confidence intervals)
☐	☒	For null hypothesis testing, the test statistic (e.g. *F*, *t*, *r*) with confidence intervals, effect sizes, degrees of freedom and *P* value noted<br>*Give P values as exact values whenever suitable.*
☒	☐	For Bayesian analysis, information on the choice of priors and Markov chain Monte Carlo settings
☒	☐	For hierarchical and complex designs, identification of the appropriate level for tests and full reporting of outcomes
☒	☐	Estimates of effect sizes (e.g. Cohen's *d*, Pearson's *r*), indicating how they were calculated

Our web collection on statistics for biologists contains articles on many of the points above.

Software and code

Policy information about availability of computer code

Data collection	No software was used to collect data
Data analysis	All softwares and statistic tests used (described in the Methods) including: FastQC (v0.11.9), BWA (v0.7.15-r1140), Sambamba (v0.6.8), inGAP-family (v1.0.0), MADpattern, Mosdepth (v0.2.7), HISAT2 (v2.1.0), StringTie (v2.0.6), and R packages randomForest (v4.6-14) for the machine-learning analysis and ggplot2 (v3.3.5) for the most of the visualization.

For manuscripts utilizing custom algorithms or software that are central to the research but not yet described in published literature, software must be made available to editors and reviewers. We strongly encourage code deposition in a community repository (e.g. GitHub). See the Nature Portfolio guidelines for submitting code & software for further information.

Data

Policy information about availability of data

All manuscripts must include a data availability statement. This statement should provide the following information, where applicable:

- Accession codes, unique identifiers, or web links for publicly available datasets
- A description of any restrictions on data availability
- For clinical datasets or third party data, please ensure that the statement adheres to our policy

The list of identified COs in the female and male populations of wild type, HEI10oe, zyp1, recq4, zyp1 HEI10oe, recq4 HEI10oe, zyp1 recq4, zyp1 recq4 HEI10oe and zyp1 recq4 HEI10oe figl1 can be accessed in Supplementary Data 1. The number of COs, sequencing depth and source of each individual sample is provided in the Source data file of Figure 1. The raw sequencing data generated in this study have been deposited in the ArrayExpress EMBL-EBI database under accession codes E-

MTAB-14424, E-MTAB-14425, E-MTAB-14426, E-MTAB-14427, E-MTAB-14428 and E-MTAB-14430. Source data are provided with this paper.

Research involving human participants, their data, or biological material

Policy information about studies with human participants or human data. See also policy information about sex, gender (identity/presentation), and sexual orientation and race, ethnicity and racism.

Reporting on sex and gender	*Use the terms sex (biological attribute) and gender (shaped by social and cultural circumstances) carefully in order to avoid confusing both terms. Indicate if findings apply to only one sex or gender; describe whether sex and gender were considered in study design; whether sex and/or gender was determined based on self-reporting or assigned and methods used.<br>Provide in the source data disaggregated sex and gender data, where this information has been collected, and if consent has been obtained for sharing of individual-level data; provide overall numbers in this Reporting Summary. Please state if this information has not been collected.<br>Report sex- and gender-based analyses where performed, justify reasons for lack of sex- and gender-based analysis.*
Reporting on race, ethnicity, or other socially relevant groupings	*Please specify the socially constructed or socially relevant categorization variable(s) used in your manuscript and explain why they were used. Please note that such variables should not be used as proxies for other socially constructed/relevant variables (for example, race or ethnicity should not be used as a proxy for socioeconomic status).<br>Provide clear definitions of the relevant terms used, how they were provided (by the participants/respondents, the researchers, or third parties), and the method(s) used to classify people into the different categories (e.g. self-report, census or administrative data, social media data, etc.)<br>Please provide details about how you controlled for confounding variables in your analyses.*
Population characteristics	*Describe the covariate-relevant population characteristics of the human research participants (e.g. age, genotypic information, past and current diagnosis and treatment categories). If you filled out the behavioural & social sciences study design questions and have nothing to add here, write "See above."*
Recruitment	*Describe how participants were recruited. Outline any potential self-selection bias or other biases that may be present and how these are likely to impact results.*
Ethics oversight	*Identify the organization(s) that approved the study protocol.*

Note that full information on the approval of the study protocol must also be provided in the manuscript.

Field-specific reporting

Please select the one below that is the best fit for your research. If you are not sure, read the appropriate sections before making your selection.

- [x] Life sciences
- [ ] Behavioural & social sciences
- [ ] Ecological, evolutionary & environmental sciences

For a reference copy of the document with all sections, see nature.com/documents/nr-reporting-summary-flat.pdf

Life sciences study design

All studies must disclose on these points even when the disclosure is negative.

Sample size	Sample size for sequencing was predetermine taking into account sequencing costs and aiming at ~200 per population, which according to the authors experience allows accurate analysis of crossover distribution along chromosomes. For cytology we aimed for 20 cells per sample; The exact number may vary depending on the difficulty of the preparation (e.g in female).
Data exclusions	No data was excluded
Replication	All attempts of replication were successful.
Randomization	*Describe how samples/organisms/participants were allocated into experimental groups. If allocation was not random, describe how covariates were controlled OR if this is not relevant to your study, explain why.*
Blinding	The investigators were not blinded.

Reporting for specific materials, systems and methods

We require information from authors about some types of materials, experimental systems and methods used in many studies. Here, indicate whether each material, system or method listed is relevant to your study. If you are not sure if a list item applies to your research, read the appropriate section before selecting a response.

Materials & experimental systems

n/a	Involved in the study	
	x	Antibodies
x		Eukaryotic cell lines
x		Palaeontology and archaeology
x		Animals and other organisms
x		Clinical data
x		Dual use research of concern
	x	Plants

Methods

n/a	Involved in the study	
x		ChIP-seq
x		Flow cytometry
x		MRI-based neuroimaging

Antibodies

Antibodies used	Three primary antibodies were used for MLH1 foci number counting: anti-REC8 raised in rat (laboratory code PAK036, dilution 1:250), anti-MLH1 raised in rabbit (PAK017, 1:200), and anti-HEI10 raised in chicken (PAK046, 1:5,000).
Validation	For each antibody, specificity was tested by checking the absence of signal in the corresponding null mutant.

Dual use research of concern

Policy information about dual use research of concern

Hazards

Could the accidental, deliberate or reckless misuse of agents or technologies generated in the work, or the application of information presented in the manuscript, pose a threat to:

No	Yes	
x		Public health
x		National security
x		Crops and/or livestock
x		Ecosystems
x		Any other significant area

Experiments of concern

Does the work involve any of these experiments of concern:

No	Yes	
x		Demonstrate how to render a vaccine ineffective
x		Confer resistance to therapeutically useful antibiotics or antiviral agents
x		Enhance the virulence of a pathogen or render a nonpathogen virulent
x		Increase transmissibility of a pathogen
x		Alter the host range of a pathogen
x		Enable evasion of diagnostic/detection modalities
x		Enable the weaponization of a biological agent or toxin
x		Any other potentially harmful combination of experiments and agents

Plants

Seed stocks

The following Arabidopsis lines were used in this study: Wild type Col-0(186AV1B4) and Ler–1 (213AV1B1) from the Versailles stock center (http://publiclines.versailles.inra.fr/). This study used zyp1-1 in Col and zyp1-6 in Ler, recq4a in Col (recq4a-4, N419423), recq4a-W387X in Ler, recq4b in Col (recq4b-2, N511330), figl1-19 in Col (SALK_089653), figl1-12 in Ler 23 and mus81-2 in Col. The HEI10 over-expression line is Col HEI10 line C2 .

Novel plant genotypes

The combinations of different mutants (Col/Ler hybrids) were obtained by a series of crossing schemes (Table S2 and Fig. S1-4).

Authentication

Describe any authentication procedures for each seed stock used or novel genotype generated. Describe any experiments used to assess the effect of a mutation and, where applicable, how potential secondary effects (e.g. second site T-DNA insertions, mosiacism, off-target gene editing) were examined.
